# Microfluidic Sensors Integrated with Smartphones for Applications in Forensics, Agriculture, and Environmental Monitoring

**DOI:** 10.3390/mi16070835

**Published:** 2025-07-21

**Authors:** Tadsakamon Loima, Jeong-Yeol Yoon, Kattika Kaarj

**Affiliations:** 1 School of Biology, Institute of Science, Suranaree University of Technology, Nakhon Ratchasima 30000, Thailand; tadsakamon.n@gmail.com; 2 Department of Biomedical Engineering, The University of Arizona, Tucson, AZ 85721, USA; jyyoon@arizona.edu

**Keywords:** AI-driven analysis, forensic analysis, microfluidic chip, smartphone integration

## Abstract

The demand for rapid, portable, and cost-effective analytical tools has driven advances in smartphone-based microfluidic sensors. By combining microfluidic precision with the accessibility and processing power of smartphones, these devices offer real-time and on-site diagnostic capabilities. This review explores recent developments in smartphone-integrated microfluidic sensors, focusing on their design, fabrication, smartphone integration, and analytical functions with the applications in forensic science, agriculture, and environmental monitoring. In forensic science, these sensors provide fast, field-based alternatives to traditional lab methods for detecting substances like DNA, drugs, and explosives, improving investigation efficiency. In agriculture, they support precision farming by enabling on-demand analysis of soil nutrients, water quality, and plant health, enhancing crop management. In environmental monitoring, these sensors allow the timely detection of pollutants in air, water, and soil, enabling quicker responses to hazards. Their portability and user-friendliness make them particularly valuable in resource-limited settings. Overall, this review highlights the transformative potential of smartphone-based microfluidic sensors in enabling accessible, real-time diagnostics across multiple disciplines.

## 1. Introduction

The growing need for rapid, portable, and cost-effective analytical tools has driven significant advancements in sensor technology across various fields. The technological advancements and innovations include the miniaturization of laboratory instruments into handheld devices [1], the use of inexpensive materials (e.g., paper, polymer, or cardboard) to reduce production costs, and the integration of built-in power, cameras, sensors, and computing capabilities into the device. Microfluidics has emerged as a miniaturized, inexpensive analytical platform. Smartphones have also emerged as versatile analytical hubs, incorporating power, cameras, sensors, and computing capabilities that can be integrated into microfluidic devices. As such, smartphone-based microfluidic sensors have gained attention for their potential to revolutionize on-site testing and diagnostics. By combining the precision of microfluidics with the accessibility and computational power of smartphones, these systems offer an adaptable and affordable solution for real-time analysis in various applications, including forensic science, agriculture, and environmental monitoring [2], with high sensitivity and selectivity in detection [3,4].

In forensic science, traditional laboratory-based methods have been employed, such as the polymerase chain reaction (PCR) for DNA analysis and high-performance liquid chromatography (HPLC) for the detection of chemical substances (e.g., illicit drugs or explosives). These methods are often time-consuming and require sophisticated equipment. Smartphone-based microfluidic sensors offer a promising alternative, enabling field-based detection and analysis at crime scenes, among other applications [5]. These sensors not only improve the speed and accuracy of forensic investigations but also enhance accessibility in resource-limited settings.

Agriculture, a sector increasingly reliant on precision monitoring, also stands to benefit from the integration of smartphone-based sensors. Farmers and agricultural professionals can use these devices to measure soil nutrients, assess water quality, and monitor plant health. The portability and user-friendliness of smartphone sensors enable on-demand analysis, reduce the time between sampling and decision-making, and facilitate better resource management, leading to enhanced crop productivity. Conventional laboratory-based testing, however, requires samples to be delivered to the laboratory, resulting in a substantial time gap between sample collection and the answer [6].

In the environmental sector, monitoring pollutants and contaminants in air, water, and soil is crucial for ensuring the health of ecosystems and the well-being of the public. Conventional methods for monitoring air, water, and soil pollution employ laboratory-based techniques, such as chromatography (gas and liquid), spectrophotometry (infrared, Raman, electrical impedance, mass, etc.), and microscopy (electron, atomic force, fluorescence, etc.), which necessitate sample collection, transportation, and skilled personnel. These methods are often time-consuming, equipment-intensive, and unsuitable for real-time monitoring. Smartphone-based microfluidic sensors provide a real-time, on-site alternative, enabling timely detection and facilitating rapid intervention in environmental hazards [7].

Therefore, this review explores recent advancements in smartphone-based microfluidic sensors, focusing on the development of microfluidic-based sensors, the integration of a smartphone into the sensor, and on-board smartphone analysis, with applications in forensic, agricultural, and environmental fields, where rapid diagnostics are required. Since the use of smartphone-based microfluidic sensors for forensics, agriculture, and environmental monitoring is not yet fully mature, regulatory certifications have not been extensively investigated. In addition, the detection limits of these sensors may not be sufficient to monitor extremely low levels of contamination. By examining the technological advancements, practical applications, and future potential of these sensors, this review aims to highlight their transformative impact on portable diagnostics and real-time analysis across multiple disciplines.

## 2. Recent Advancements in Fabrication of Microfluidic-Based Sensors

Microfluidics is the manipulation of small volumes of fluids through micro-scale channels, often integrated onto compact platforms known as lab-on-a-chip systems [8]. At this scale, fluid behavior is governed by unique physical principles, such as strict laminar flow due to low Reynolds numbers (requiring diffusion-based mixing), the dominance of surface tension over gravity (leading to capillary action), and the electrokinetic effects that can drive fluid motion [9]. These phenomena allow for precise control of fluid movement, mixing, and reactions, making microfluidic systems highly efficient for chemical and biological analytical assays [10].

Recent advancements and innovations in microfluidic fabrication have significantly enhanced analytical capabilities. These developments enable the creation of precise, miniaturized platforms that integrate complex sensing functions, offering enhancing performance for forensic, agricultural, and environmental monitoring applications, with improved portability and cost-effectiveness.

### 2.1. Chip Design

**Figure 1:**
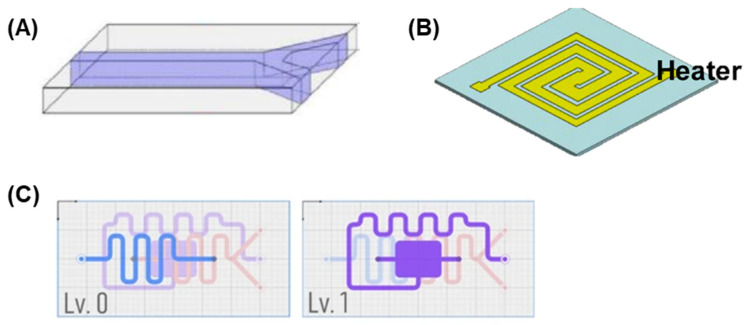
Various microfluidic chip geometries and designs. (**A**) Integration of microfluidic platforms with a Y-shaped straight channel. This design was used for a terahertz time-domain spectroscopy (THz-TDS) system [12]. (**B**) Spiral-shaped microfluidic platforms for the miniaturized PCR system to detect plant pathogens. Adapted from [13] under Creative Commons Attribution License. (**C**) Open-source interactive design platform for 3D-printed microfluidic devices. Reproduced from [14] under Creative Commons Attribution License.

The development of microfluidic chips is an interdisciplinary task that involves principles from fluid dynamics, biochemistry, and engineering, with the main goal being designing systems that offer efficient fluid operations. This process involves creating fluid channels; embedding sensing elements; integration with components such as valves, pumps, and detection systems; as well as high performance accuracy and reliability. The conceptualization of microfluidic chips encompasses substantial consideration of fluid dynamics principles, with particular attention to microscale flow behaviors and capillary phenomena. The chip designs rely upon specialized software instruments, notably AutoCAD, SolidWork, and COMSOL Multiphysics, which provide essential functionalities for geometric modeling and fluid behavior simulation [11]. Additional software tools contribute complementary capabilities throughout the design workflow, enabling comprehensive chip architecture development and performance prediction prior to fabrication processes. The design considerations are as follows:

- **Channel geometry:** The dimensions and layout of microchannels are crucial for controlling fluid flow, mixing, and reaction kinetics. For example, straight channels are employed for simple flow control (Figure 1A), while serpentine channels are often used to enhance mixing (Figure 1B,C) [12].
- **Integration of functional components:** Modern microfluidic chips often incorporate components such as valves, pumps, and sensors to enable complex operations. For instance, electrochemical sensors can be integrated into microfluidic channels for real-time detection of analytes [13].
- **Portability and scalability:** Designs must balance portability for field applications with scalability for mass production. Recent advancements in modular designs allow for customizable and reusable chips (Figure 1C) [14].

Microfluidic chips provide significant advantages in forensic applications through their ability to process minute sample volumes with rapidity and precision. These devices are particularly valuable for forensic DNA analysis, where they incorporate integrated PCR (polymerase chain reaction) chambers for DNA amplification [15]. Such integration necessitates sophisticated thermal regulation and fluid handling systems. The design typically incorporates electrophoresis channels alongside the PCR functionality, creating comprehensive analytical platforms for forensic investigations. The forensic implementation of these chips prioritizes rapid processing timeframes and enhanced sensitivity parameters to accommodate the analysis of degraded evidence or samples with low concentration levels.

Developing a microfluidic device involves optimizing mobility for forensic and agricultural applications. Fluid dynamics principles must be applied throughout the process. Microfluidic chips also need to be designed to simplify operations, cost-effective production, and integrate detection methods, such as fluorescence or mass spectrometry.

### 2.2. Materials

The material selection criteria encompass biocompatibility specifications, optical property parameters, chemical resistance characteristics, mechanical durability requirements, and sensor integration capabilities. Different substrate materials present distinct advantage profiles necessitating comprehensive evaluation within the context of intended implementation scenarios. The commonly used materials are polymers, glass, silicon, paper, and conductive materials [16].

#### 2.2.1. Polymers

Polydimethylsiloxane (PDMS) is one of the most commonly used materials in microfluidics due to its excellent transparency, ease of fabrication, and flexibility [16,17]. PDMS is ideal for biological and forensic applications, particularly for DNA amplification and detection, as it allows for easy bonding with glass or silicon and is chemically inert. However, PDMS has limitations such as its susceptibility to the adsorption of proteins and other biological molecules, which can interfere with sensing applications [18].

Other polymeric materials, such as polymethylmethacrylate (PMMA) and polystyrene, have also gained popularity in microfluidic chip fabrication. These materials are durable, relatively inexpensive, and compatible with injection molding and other mass-production techniques [19]. PMMA, for instance, is often used in agricultural applications for detecting pesticides or in environmental applications for detecting nutrients in soil samples due to its optical transparency and chemical resistance [20]. Limitations of PDMS and PMMA include low stiffness and gas permeability. Alternative polymers, such as cyclic olefin copolymer (COC), composed of cyclic olefin monomers (norbornene) and linear olefins (ethylene), offer improved properties, including low autofluorescence, thermal resistance, and enhanced biocompatibility [21].

#### 2.2.2. Glass and Silicon

Glass is a traditional material for microfluidic chip fabrication due to its excellent chemical stability, optical transparency, and compatibility with various detection methods, including fluorescence and electrochemical sensors. Glass chips are often used in applications where precise control of fluid flow and high sensitivity are required, such as in forensic DNA analysis and environmental monitoring of chemical pollutants [22,23]. However, glass fabrication is more expensive and challenging compared to PDMS.

Silicon-based microfluidic chips offer high precision and scalability, making them ideal for industrial-scale applications. Silicon chips are often used for applications that require robust, high-performance systems, such as environmental pollutant monitoring, such as heavy metals and pesticides, and forensic analysis. In contrast, the manufacturing of microfluidic devices that incorporate glass and silicon requires a cleanroom facility, thereby raising the costs of fabrication. Moreover, it necessitates the use of toxic chemicals, including hydrofluoric acid, limiting their widespread use [24,25].

#### 2.2.3. Paper

Paper-based microfluidic devices are gaining popularity due to their low cost, portability, and ease of use. They are particularly suitable for point-of-care testing and field applications [26,27]. Paper-based chips are used for the rapid detection of plant pathogens and environmental contaminants [28].

#### 2.2.4. Conductive Materials

For the purpose of integrating sensors and detection methods, such as electrochemical and impedance sensors, conductive materials, including gold, platinum, and carbon-based materials like graphene, are regularly incorporated into microfluidic chips. These materials are indispensable for the detection and quantification of chemical or biological analytes in forensic, agricultural, and environmental contexts [29,30].

In conclusion, the fabrication of microfluidic sensors for forensic, agricultural, and environmental applications requires careful consideration of chip design, fabrication techniques, and material selection. Advances in chip design have enabled the integration of multiple sensing elements and detection methods into compact, portable platforms that can be deployed in diverse field settings. The continued development of fabrication techniques, particularly soft lithography, injection molding, and 3D printing, has made it easier to produce microfluidic sensors with high precision and low cost. As materials science progresses, new materials are being explored to enhance the performance and versatility of microfluidic devices. Moving forward, the continued refinement of fabrication techniques and material innovations will drive the next generation of microfluidic sensors, expanding their applications and impact in forensic, agricultural, and environmental monitoring.

### 2.3. Fabrication Techniques

**Figure 2:**
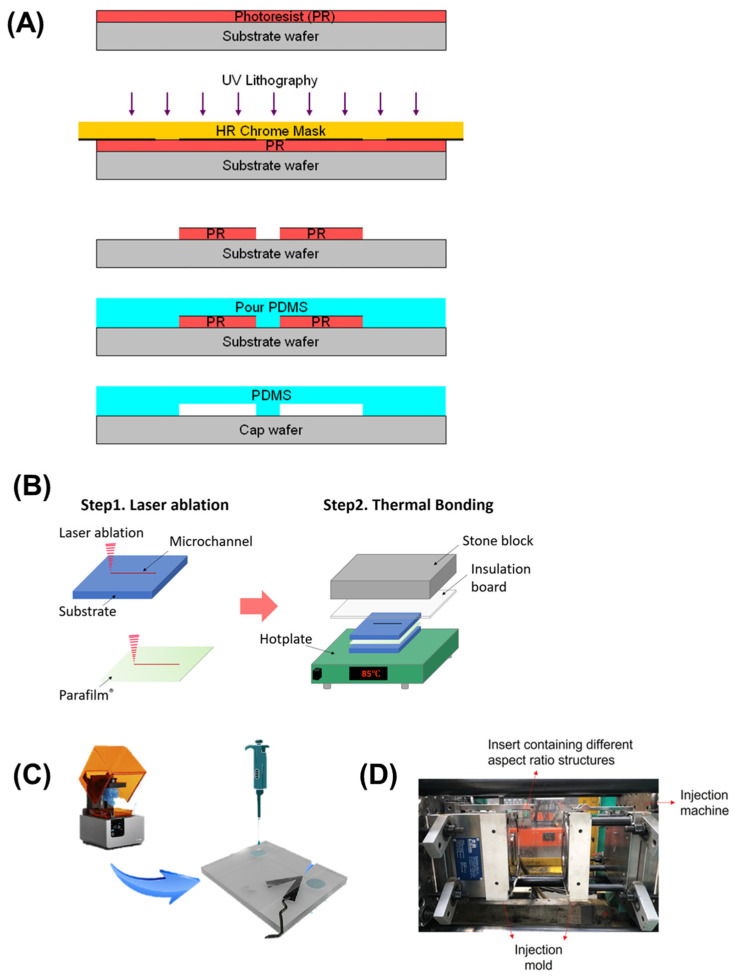
Microfluidic chip fabrication techniques. (**A**) Soft lithography using PDMS (polydimethylsiloxane). Adapted with permission from [33]. (**B**) Laser ablation. Reproduced with permission from [36]. (**C**) 3D printing. Adapted with permission from [38]. (**D**) Injection molding. Reproduced with permission from [45].

The fabrication of microfluidic chips involves various techniques that can be tailored to the specific requirements of the intended application, each with advantages and limitations. Recent advancements have focused on improving precision, reducing costs, and enabling large-scale production. Some of the most commonly used techniques for microfluidic device fabrication include soft lithography, laser ablation, 3D printing, and injection molding [31,32], which are illustrated graphically in Figure 2.

#### 2.3.1. Lithographic Techniques

Soft lithography, particularly using polydimethylsiloxane (PDMS), is the most widely used for fabricating microfluidic chips. It involves creating a mold from a master wafer using photolithography, followed by the casting of elastomeric materials such as polydimethylsiloxane (PDMS) to form the microfluidic channels (Figure 2A) [33]. Soft lithography is favored for its high precision, low cost, flexibility, transparency, biocompatibility, and ability to create complex channel structures [34,35].

#### 2.3.2. Laser Ablation

Laser ablation involves using focused laser beams to etch or ablate the surface of materials such as glass or polymers, creating microfluidic channels (Figure 2B). This technique enables the fabrication of high-precision chips, especially when traditional photolithography is impractical due to material instability, high costs, and the time-consuming nature of the process [36]. Laser ablation is increasingly being used in applications requiring rapid prototyping of microfluidic devices with complex geometries, such as environmental sensors for pollutant detection [37].

#### 2.3.3. Three-Dimensional Printing

The 3D printing methodology represents an emerging fabrication paradigm for microfluidic sensing platforms, enabling three-dimensional structural realization with enhanced spatial resolution parameters. This approach demonstrates particular utility for complex configurations requiring precise geometric specifications (Figure 2C). Techniques such as stereolithography (SLA) and fused deposition modeling (FDM) are used to create complex 3D structures with high precision [38]. This technique can be beneficial for designing custom chips for forensic and environmental applications, where the need for unique geometries and high precision is paramount. This strategy offers significant advantages, including economic efficiency, accelerated prototyping, and multi-layer integration [39]. A 3D-printed electrochemical microfluidic platform with bismuth-modified electrodes enables sequential stripping analysis for the detection of lead and cadmium in environmental monitoring applications [40]. Two-photon polymerization (2PP) is an advanced 3D microfabrication technique that uses a tightly focused femtosecond laser to induce localized polymerization within a photosensitive material. This nonlinear process enables the creation of highly precise, complex microstructures with sub-micron resolution, making it ideal for fabricating intricate features in microfluidic chips that are challenging to achieve with traditional lithography methods [41,42]. 3D-printed microfluidic chips are being utilized more frequently in the field of environmental monitoring, necessitating tailored designs for particular applications [43].

#### 2.3.4. Injection Molding and Casting

Injection molding methodology constitutes a high-throughput fabrication approach wherein molten thermoplastic substances are introduced into predefined molds for microfluidic structural formation (Figure 2D) [44]. This technique is significant for mass-production scenarios requiring substantial device quantity and enhanced uniformity parameters. The process exhibits considerable relevance for disposable sensing platforms within agricultural and environmental monitoring contexts due to its pronounced scalability characteristics and economic efficiency advantages [45]. These attributes position injection molding as an optimal fabrication strategy for smartphone-integrated microfluidic sensing applications requiring widespread deployment across diverse implementation domains.

In conclusion, selecting a suitable fabrication technique for microfluidic sensors largely depends on the specific requirements of each application domain—namely forensic science, agriculture, and environmental monitoring. In forensics, where rapid prototyping and portability are essential, techniques like laser ablation offer flexibility and precision without the need for cleanroom facilities, making them ideal for developing field-deployable devices for preliminary screening [46]. For agricultural applications, low-cost, scalable methods such as soft lithography and paper-based microfluidics are favored due to their ease of mass production and disposability, which are key for frequent on-site testing of pesticides, pathogens, and soil nutrients [47]. In environmental monitoring, where high resolution and sensitivity are crucial for detecting trace pollutants, advanced methods like two-photon polymerization (2PP) enable the creation of complex, high-precision 3D microstructures tailored for sophisticated sensing tasks [48]. Each technique offers unique advantages, and the balance between performance, cost, and deployment conditions shapes their suitability.

## 3. Microfluidic Sensors for Forensic Applications

Forensic science has experienced significant transformation through smartphone-integrated microfluidic sensing technologies, providing rapid and sensitive analytical methods for biological and chemical substance evaluation at crime scenes [49]. These innovations address critical limitations in conventional forensic approaches, including sample shortage problems, contamination risks, and time-sensitive analysis requirements. Traditional forensic procedures require evidence collection, laboratory transportation, and extensive analytical processes that delay investigations. Conventional methods typically need large sample volumes, specialized equipment, and highly trained personnel, creating operational delays within criminal justice systems [50]. Microfluidic integration significantly reduces these procedure limitations while improving analytical efficiency [51].

### 3.1. Crime Scene Analysis

**Figure 3:**
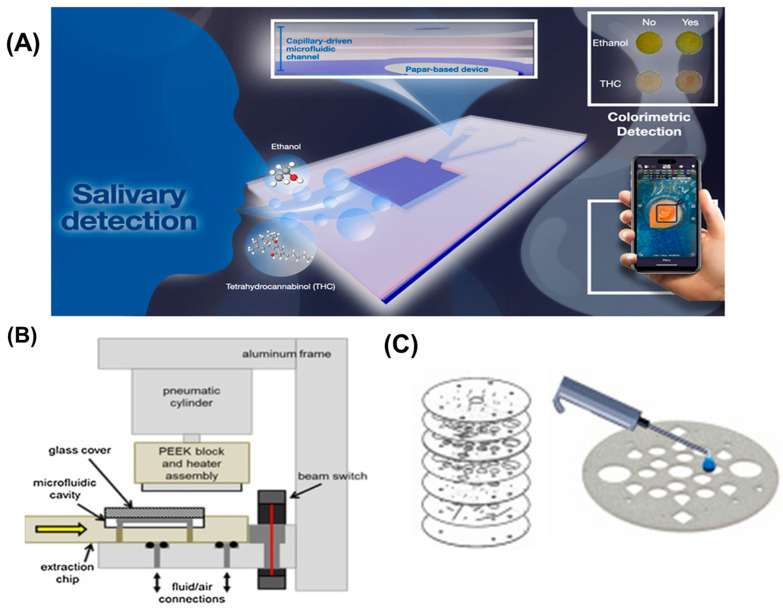
Microfluidic sensors for forensic analysis. (**A**) Paper-based device integrated with smartphone for colorimetric salivary detection of ethanol and Δ^9^-tetrahydrocannabinol (THC). Reproduced with permission from [54]. (**B**) Automated microfluidics extraction system coupled with Q-TOF mass spectrometer for direct analysis of textile dyes from trace fibers (yellow arrow represented the chip insertion point to the device). Reproduced with permission from [58]. (**C**) Automated solid phase DNA extraction on a lab-on-a-disk for DNA analysis and profiling. Reproduced with permission from [67].

The ability to conduct on-site crime scene testing of biological samples, such as blood or saliva, is crucial for timely investigations. Microfluidic devices can be engineered to identify particular biomarkers or drugs, which allows forensic investigators to efficiently obtain crucial evidence [52,53]. For instance, a microfluidic device integrated into a smartphone can analyze blood samples for alcohol or drug levels, providing instant results that can guide investigative decisions. Srisomwat et al. developed microfluidic colorimetric paper analytical device (μCD-PAD) enables simultaneous ethanol and THC detection in viscous saliva samples using smartphone-based real-time analysis without sample preparation or cross-interference (Figure 3A) [54]. Utilizing a colorimetric technique, these devices enable simple analysis using smartphone cameras, thereby ensuring they are accessible and cost-efficient for immediate forensic screening.

### 3.2. Trace Evidence Analysis

Trace evidence analysis has benefited significantly from microfluidic approaches that maximize information extraction from limited sample volumes [55]. Conventional techniques for examining gunshot residue, explosives, and trace materials require sophisticated sample preparation and instrumentation, such as scanning electron microscopy with energy-dispersive X-ray spectroscopy or gas chromatography–mass spectrometry [56,57]. Sultana et al. have investigated an automated microfluidics–mass spectrometry system that rapidly extracts and differentiates multiple dyes from millimeter-length fiber threads within twelve minutes for forensic analysis (Figure 3B) [58].

### 3.3. Blood Pattern Analysis

Blood pattern analysis has traditionally relied on visual interpretation and geometric reconstruction, with limited quantitative approaches [59,60]. Microfluidic technologies have introduced novel capabilities for characterizing blood rheology, aging, and biological profile. Microfluidic devices measure blood viscosity changes that occur during coagulation and aging, potentially establishing more accurate timelines in criminal investigations [61]. The controlled fluid dynamics within microchannels enables systematic study of blood droplet formation, trajectory, and impact patterns under various conditions, enhancing the scientific foundation of bloodstain pattern analysis [62].

### 3.4. DNA Analysis

DNA analysis represents one of the most significant applications of microfluidic technology in forensic science [63]. Traditional DNA profiling involves multi-step processes including extraction, quantification, amplification, and electrophoretic separation, typically requiring 24–72 h in centralized laboratories [64]. Microfluidic platforms have revolutionized this workflow by integrating these steps onto a single chip, reducing analysis time to 1–2 h while minimizing contamination risks and sample consumption [65].

Chip-based DNA extraction utilizes various mechanisms including solid-phase extraction on silica surfaces, magnetic bead-based isolation, and isotachophoresis for purification from complex forensic samples such as blood, saliva, and touch DNA (Figure 3C) [66,67]. These approaches significantly outperform traditional phenol–chloroform extraction or commercial spin column kits in terms of processing time and integration potential [68].

Microfluidic PCR has addressed limitations of conventional thermal cycling through designs that reduce thermal mass and optimize heat transfer, enabling rapid amplification of target sequences from trace samples [69]. Novel approaches include digital PCR on microfluidic platforms, providing absolute quantification capabilities particularly valuable for mixed or degraded samples commonly encountered at crime scenes [70].

The integration of capillary electrophoresis on microfluidic chips has enabled rapid separation and detection of amplified fragments, completing the DNA profiling workflow within a unified system [71]. Smartphone-based detection of fluorescently labeled DNA fragments has made field-deployable genetic analysis possible, potentially revolutionizing crime scene investigation protocols [72].

### 3.5. Illicit Drug Detection

The rapid detection of illicit substances is critical in forensic investigations. Smartphone-based microfluidic sensors can identify drugs through colorimetric or electrochemical methods, providing law enforcement with immediate, actionable information from a presumptive test [73,74]. These devices can also be used for drug testing in various settings, including roadside testing for impaired driving. Bruijns et al. have developed a cyclic olefin copolymer (COC) microfluidic device to enable rapid forensic crime scene testing through chemical-resistant, optically transparent chips, supporting forensically significant reactions involving COC chips, specifically the spot test for the analysis of illicit drugs and the amplification of DNA [75].

### 3.6. Toxicological Screening

Toxicological analysis represents another critical application of microfluidic technology in forensic science [76]. Conventional toxicology screening involves immunoassay-based presumptive testing followed by confirmatory analysis using chromatography coupled with mass spectrometry, requiring specialized laboratory infrastructure and considerable analysis time [77].

Microfluidic immunoassays have streamlined toxicological screening by miniaturizing reaction volumes and accelerating diffusion-limited binding kinetics [78]. These platforms enable simultaneous detection of multiple drugs of abuse from biological samples including urine, blood, and oral fluid, with sensitivity comparable to laboratory-based methods [79]. On-chip chromatographic separations have enabled field-deployable confirmatory testing, addressing limitations of presumptive screening methods [80]. Microfluidic devices capable of sample preparation followed by separation and detection provide comprehensive toxicological analysis without laboratory infrastructure. Microfluidic platforms enable the chemical profiling of minute particles through controlled reactions within defined microchannels. These systems facilitate colorimetric, electrochemical, or fluorescence-based detection compatible with smartphone imaging, providing rapid presumptive testing at crime scenes. Despite the advantages, challenges remain in achieving the required sensitivity and specificity for forensic applications. Additionally, the need for training personnel to use these devices and interpret results accurately can hinder widespread adoption.

## 4. Microfluidic Sensors for Agricultural and Environmental Applications

In agriculture, smartphone-based microfluidic sensors are transforming how farmers monitor crop health, soil conditions, and environmental factors. Environmental monitoring is another area where smartphone-based microfluidic sensors are making significant contributions, enabling real-time assessments of pollutants and ecosystem health.

### 4.1. Plant Pathogen Detection

Plant disease diagnosis has historically relied on visual symptoms, culture-based isolation, or laboratory-based molecular techniques such as PCR and ELISA, resulting in delayed intervention and consequent crop losses [81]. Traditional approaches require specialized expertise for symptom interpretation or complex equipment for molecular confirmation, limiting their deployment in agricultural settings [82].

The advent of microfluidic nucleic acid amplification tests has greatly enhanced the ability to detect plant pathogens, enabling the specific identification of viral, bacterial, and fungal species directly in field environments. Loop-mediated isothermal amplification (LAMP), extensively utilized for the detection of pathogens on microfluidic chips, offers particular advantages through an isothermal operation compatible with battery-powered heating elements [17,83]. LAMP amplifies DNA under isothermal conditions with high efficiency, a more cost-effective, easy-to-use method, and ideal for on-site testing. Lab-on-a chip (LOC) devices can autonomously mix and dispense samples for detecting multiple plant viruses simultaneously via LAMP [84]. Sun et al. developed a microfluidic test platform to rapidly detect rice false smut (RFS) [85]. They combined spore purification, nucleic acid release, and amplification into one chip. A micro air pump facilitated the separation of spores from impurities, ensuring the complete collection of spores via airflow. The products of LAMP could be mixed with SYBR Green I for visual result observation. Similarly, Shymanovich et al. developed rapid nucleic acid extraction methods with microneedles and combined these with LAMP assays for early blight (*Alternaria linariae*, *A. alternata*, and *A. solani*) and bacterial spot of tomato (*Xanthomonas perforans*) pathogen detection in the field [86].

**Figure 4:**
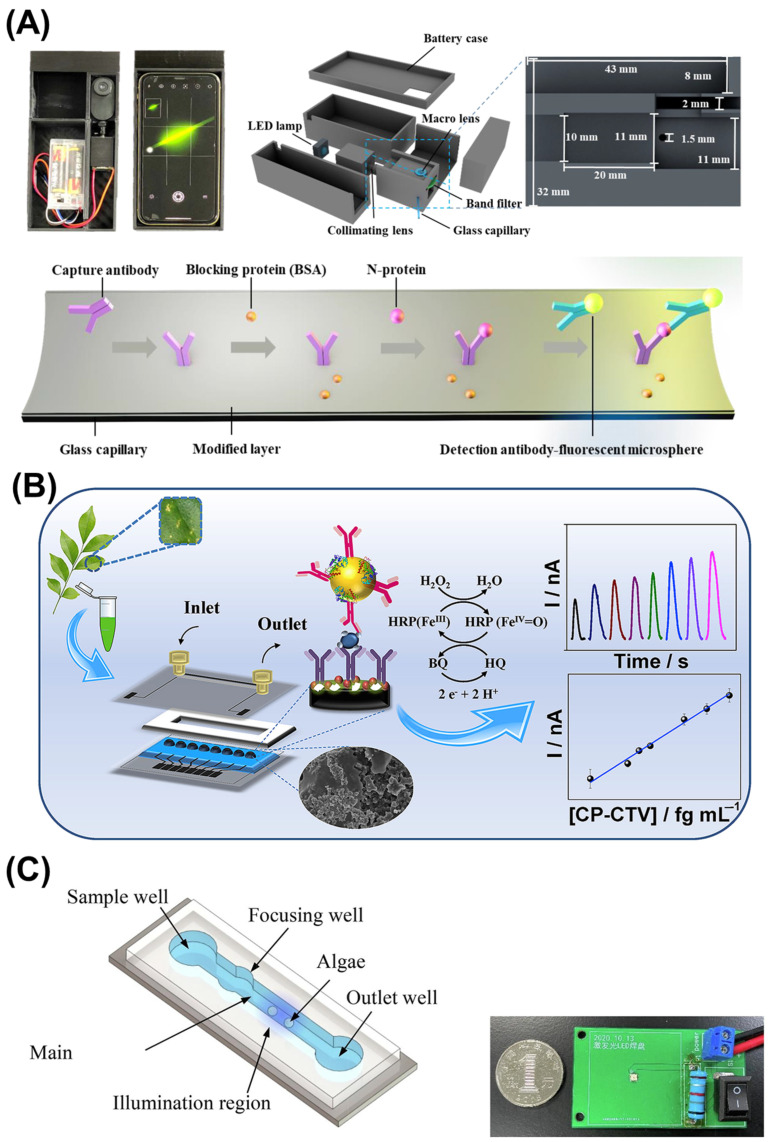
Microfluidic sensors for agricultural and environmental applications. (**A**) Smartphone-based rapid immunoassay of biomarkers. Reproduced with permission from [88]. (**B**) Electrochemical immunoassay of citrus tristeza virus from citrus samples. Reproduced with permission from [89]. (**C**) Smartphone-based real-time detection, counting, and sizing of living algae. Reproduced with permission from [103].

Immunological detection of plant pathogens has been miniaturized onto microfluidic platforms, utilizing controlled fluid handling to enhance sensitivity and reduce analysis time [87]. These approaches typically employ colorimetric or fluorescent readouts detectable by smartphone cameras, providing quantitative results that inform treatment decisions (Figure 4A) [88]. The development of microfluidic immunosensors incorporating micromagnetic beads alongside carbon-based screen-printed electrodes has been achieved for the rapid and accurate detection of fungal pathogens like *Botrytis cinerea*. These sensors can detect pathogens even in asymptomatic fruits. In addition, Freitas et al. developed a rapid detection methodology for the *Citrus tristeza* virus (Figure 4B) [89]. The immunosensor system utilizes magnetic beads decorated with horseradish peroxidase enzyme and polyclonal capture antibody for ultrasensitive capsid protein detection through disposable microfluidic electrochemical devices. This analytical platform presents promising potential for multiplex pathogen detection applications in plant diagnostic contexts.

### 4.2. Soil Analysis

Soil health assessment traditionally requires extensive laboratory analysis of physical, chemical, and biological parameters using techniques such as atomic absorption spectroscopy, ion chromatography, and culture-based microbial enumeration [90]. These approaches necessitate sophisticated equipment, trained personnel, and substantial sample preparation, limiting their accessibility to farmers and field researchers [91].

Microfluidic platforms have transformed soil nutrient analysis by enabling rapid quantification of essential elements including nitrogen, phosphorus, and potassium directly in field settings [92]. Traditional colorimetric assays have been adapted to microfluidic formats, with smartphone cameras serving as detectors that provide quantitative results comparable to laboratory instrumentation [93]. Khongpet et al. developed a microfluidic hydrodynamic sequential injection platform utilizing laser engraving fabrication on acrylic substrate materials [94]. The system demonstrated effective phosphate determination capabilities in soil sample analysis while exhibiting automated operation, compact design configuration, cost-effectiveness, and minimal chemical consumption characteristics for agricultural analytical applications.

Soil microbiome characterization has been revolutionized through microfluidic approaches that combine DNA extraction, amplification, and detection systems. These integrated platforms enable the identification of beneficial microorganisms, plant pathogens, and indicators of soil health without specialized laboratory infrastructure [95,96,97].

Heavy metal contamination assessment has traditionally required digestion procedures followed by atomic absorption spectroscopy or inductively coupled plasma mass spectrometry [98]. Microfluidic electrochemical sensors now permit on-site detection of toxic metals with sensitivity, enabling immediate remediation decisions [99].

### 4.3. Water Quality Assessment

Water quality monitoring traditionally involves sample collection for laboratory analysis using techniques such as atomic absorption spectroscopy for metals, chromatography for organic contaminants, and culture-based methods for microbial contamination. These approaches present significant barriers to regular monitoring, particularly in remote areas or developing regions where laboratory access is limited [98,100,101].

Irrigation plays a crucial role in agriculture, and monitoring irrigation water quality is essential for crop health. Microfluidic sensors can detect contaminants or pathogens in water sources, allowing farmers to ensure their crops receive clean and safe water. Microfluidic platforms have enabled comprehensive water quality assessment through integration of multiple detection methods on a single chip. Parameters including pH, dissolved oxygen, conductivity, and specific contaminants can be simultaneously evaluated using electrochemical, optical, or colorimetric detection methods compatible with smartphone readout systems [102]. Tong Hou et al. highlighted the use of a smartphone-integrated microfluidic platform for ballast water analysis that incorporates automated algorithms for living algae detection, counting, and sizing, demonstrating detection accuracy exceeding 92% for environmental monitoring applications (Figure 4C) [103].

Microbial water quality testing has been transformed through microfluidic approaches that eliminate the need for culture-based enumeration procedures. Molecular techniques including PCR and LAMP have been adapted to microfluidic formats, enabling species-specific identification of waterborne pathogens within 30–60 min compared to 24–48 h for traditional culture methods. Continuous monitoring systems based on microfluidic technology provide temporal resolution previously unattainable with discrete sampling approaches, enabling real-time data streams for immediate intervention when parameters deviate from acceptable ranges [104].

### 4.4. Environmental Monitoring

Microfluidic sensors demonstrate significant utility in environmental monitoring applications, providing critical data for regulatory compliance and public health protection. These devices can detect various environmental pollutants in air and water matrices, with smartphone-integrated sensors capable of analyzing water samples for heavy metals or organic contaminants, enabling immediate identification of pollution sources and contamination events [105].

Biodiversity monitoring represents another essential application area for assessing ecosystem health parameters. Microfluidic platforms can identify microbial communities or detect specific species within environmental samples [102]. The integration of these devices with smartphone technology enables researchers to gather and analyze data in real time, facilitating conservation efforts and ecological assessment procedures.

Climate change research requires robust data collection methodologies to understand ecosystem impacts over temporal scales. Smartphone-based microfluidic sensors provide valuable environmental parameter data including temperature, pH, and nutrient concentration measurements, enabling researchers to study environmental trends and changes over extended time periods. This technological integration supports comprehensive environmental monitoring programs while maintaining portability and accessibility for field-based research applications, thereby enhancing the scope and effectiveness of environmental assessment and conservation initiatives [106,107,108].

### 4.5. Heavy Metal Ion Detection

Heavy metal contamination in soil and water sources poses significant environmental and health risks globally. Microfluidic sensors offer promising alternatives for rapid, portable, and cost-effective detection of heavy metals. Recent studies demonstrate various microfluidic approaches for heavy metal sensing. Electrochemical microfluidic devices using bismuth electrodes show excellent sensitivity for cadmium detection in soil pores and groundwater samples on-site [109]. Smartphone-integrated systems can also be employed for real-time monitoring applications. Xiao et al. developed a portable smartphone-based reader using fluorescent carbon nanodot paper microarrays for detecting Hg^2+^, Pb^2+^, and Cu^2+^ in river water. With minimal sample preparation, users can pipette samples, insert chips, and capture fluorescence via an app [110]. A dual-sided microfluidic device enabling single-step detection of Cu^2+^, Fe^2+^, Ni^2+^, NO_2_^−^, and PO_4_^3−^ in water samples. Using smartphone-assisted analysis and capillary flow, it provides rapid, on-site results [111]. The design addresses fluidic challenges, providing rapid, reliable, cost-effective multiplex contaminant quantitation suitable for practical field analysis in resource-limited environments.

Microfluidic sensors for heavy metal ion detection are currently focused on improving sensitivity, selectivity, and practical deployment. Future research should focus on standardization, commercial viability, and the simultaneous detection of multiple metal ions as comprehensive environmental monitoring solutions.

## 5. Smartphone-Based Detection Method on Microfluidic Sensors Used in Forensic, Agricultural, and Environmental Applications

The merging of smartphones with microfluidic sensors has significantly changed the availability and application of these devices across multiple disciplines. This section explores the methodologies involved in implementing smartphone on microfluidic chips, the underlying principles guiding these detection methods, and the advanced techniques utilized for data processing and analysis.

### 5.1. Implementation of Smartphones on Microfluidic Chips

Smartphones have become ubiquitous tools, offering advanced computational capabilities, high-resolution cameras, and a range of sensors that can be leveraged for microfluidic applications. The implementation of smartphones on microfluidic chips involves several key components.

**Hardware Integration:** The microfluidic chip must be designed to facilitate the coupling of its fluidic channels with the smartphone’s optical and electronic components. This often requires custom adapters or holders that align the chip with the smartphone camera or other sensors (Figure 4A). For example, a portable smartphone-based sensing system utilizing 3D-printed chips addresses fabrication complexity, signal capture stability, and app flexibility limitations in paper-based microfluidic technology for biochemical assays, demonstrating versatile detection capabilities for organophosphorus pesticides and fruit analysis [112]. These attachments often incorporate LED illumination, optical filters, and lenses to optimize signal-to-noise ratios for specific detection modalities [113,114].

**Optical Detection Systems:** Many microfluidic sensors utilize optical detection methods, such as fluorescence, absorbance, or colorimetric analysis. Smartphones, equipped with high-resolution cameras, can capture images that are processed to quantify the response of the sensor. For instance, in a study by Brás et al., a smartphone-based microfluidic device was developed for pathogen infection biomarker in grapes, demonstrating how the camera could capture colorimetric changes indicative of target analytes [115]. Moreover, Lee et al. have contributed to the fields of on-chip super-resolution fluorescence imaging and compact high-sensitivity optofluidic SPR sensing [116]. While the present advancements are still at the feasibility-study stage, metallic nanostructured electrodes integrated into microfluidic devices can facilitate on-chip multimodal (optical and electrical) measurements of cells cultured in microfluidic devices.

**Software Development:** Custom applications or software are essential for processing the captured images. These applications can include image processing algorithms that analyze the color intensity or other parameters to yield quantitative results. The development of user-friendly interfaces is crucial for non-expert users, especially in field applications [117]. Parker et al. have developed an open-source program called ColorScan that can detect paper-based microfluidic devices that perform colorimetric measurements require quantitative image analysis, which automatically recognizes and measures signal-containing zones from device images, regardless of output zone geometry or spatial arrangement, reducing the need for time-consuming and user-dependent image processing procedures [118].

**Power Supply and Connectivity:** The smartphone can provide power to the microfluidic device, eliminating the need for external power sources. Additionally, smartphones can facilitate data transmission via Bluetooth or Wi-Fi, allowing for remote monitoring and data sharing in real time.

Smartphones combined with microfluidic chips enhance detection via integrated hardware, optical detecting, custom software, and connectivity. Systems provide portability without sacrificing precision. Efforts towards standardization have been initiated to establish design guidelines for smartphone attachments that accommodate various device models while ensuring consistent analytical performance.

### 5.2. Working Principle of Smartphone Detection on Microfluidic Chips

The working principles of smartphone-based detection methods on microfluidic chips can be categorized primarily into optical and electrochemical detection methods.

#### 5.2.1. Optical Detection

**Figure 5:**
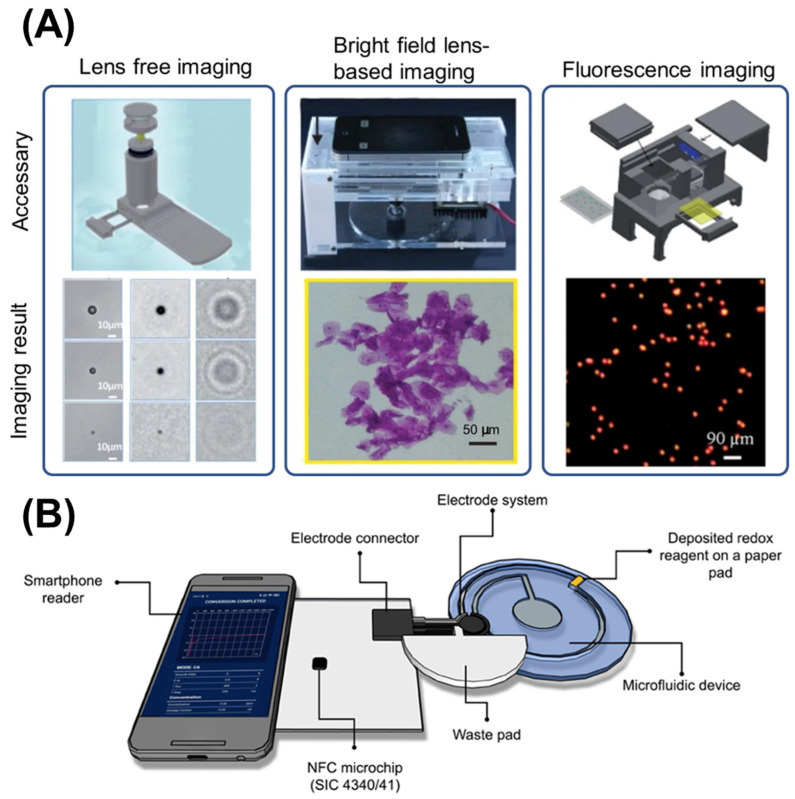
Different modalities of smartphone detection on microfluidic chips. (**A**) Optical detection is achieved by capturing images of the particles of interest and fluid flow through the microchannels. Reproduced with permission from [119]. (**B**) Electrochemical detection of a smartphone-based microfluidic device integrated with nanobody recognition for C-reactive protein. Reproduced with permission from [123].

This is a widely utilized detection approach in microfluidics that are integrated into smartphones. It usually consists of capturing images of the fluid flow through the microchannels. The detection may depend on multiple phenomena:

- **Fluorescence:** Many assays utilize fluorescent markers that emit light when excited, allowing for sensitive detection (Figure 5A) [119]. Smartphones can capture the emitted light and software can analyze the intensity of fluorescence to determine the concentration of analytes [114,119].
- **Colorimetric Detection:** Colorimetric detection offers simplicity and accessibility, requiring minimal additional components while providing quantitative analysis through RGB or HSV color space analysis [88,120]. This method relies on color changes in the fluid, which can be quantified using the smartphone camera. For example, a pH indicator dye might change color in response to pH variations, providing a visual cue that can be analyzed quantitatively.
- **Absorbance Measurement:** Similar to colorimetric methods, absorbance can be measured by capturing images of the fluid with varying concentrations of colored analytes. Algorithms can be applied to determine concentration based on the intensity of color observed in captured images [82].
- **Chemiluminescence:** Chemiluminescence methodology involves photon generation through chemical reaction processes, with bioluminescence specifically occurring within living organisms or cellular systems. Similar to colorimetric techniques, chemiluminescence readout can be captured utilizing digital cameras and smartphone platforms, incorporating computer-based operation and data processing capabilities for quantitative biomarker analysis in portable analytical applications [121,122].

#### 5.2.2. Electrochemical Detection

This method often employs electrodes integrated into the microfluidic chip that can measure changes in current or voltage in response to chemical reactions [123]. Smartphones can interface with these electrochemical sensors through additional circuitry that converts signals into a format that can be read by the phone (Figure 5B) [124,125].

#### 5.2.3. Electrochemiluminescence

This method integrates electrochemical techniques with luminescence and offers a superior detection capability for paper-based sensors, owing to the cost-effectiveness and miniaturization advantages of electrochemical methods, combined with the heightened sensitivity of luminescent techniques [126,127].

### 5.3. Image Processing Techniques

Image processing constitutes a critical component in smartphone-based microfluidic detection systems, transforming raw visual data into quantitative analytical results. Following image capture using smartphone platforms, several processing techniques are employed to extract meaningful analytical information, crucial for converting visual data into quantifiable measurements [62,128].

Preprocessing procedures typically encompass noise reduction, background correction, and region-of-interest identification to isolate relevant signal elements within captured images. Image segmentation processes involve isolating specific areas of interest, such as microfluidic channels or color changes; utilizing algorithms, including thresholding and particularly adaptive thresholding methodologies which facilitate the differentiation of areas based on color intensity variations [129]; edge detection; and region-growing techniques.

Feature extraction procedures involve obtaining various characteristics from processed images, including area measurements, shape parameters, and color intensity values. These extracted features establish essential correlations between image data and analyte concentration levels. Techniques such as Hu Moments and contour analysis may be employed for comprehensive feature characterization [130]. Colorimetric analysis algorithms extract intensity values from appropriate color channels, comparing them with calibration data to determine analyte concentrations [131]. Advanced approaches incorporate color correction using reference patches to compensate for ambient lighting condition variations. Machine learning algorithms, particularly convolutional neural networks, enhance detection accuracy through pattern recognition capabilities trained on datasets with known concentrations [132,133,134]. Automated feature extraction enables analysis of complex visual patterns including immunochromatographic test lines, droplet distributions, and cellular morphologies, providing objective quantification of results that were previously dependent on subjective visual interpretation. Software platforms such as ImageJ and OpenCV facilitate processing tasks, while custom mobile applications integrate these tools for automated and streamlined smartphone-based analysis [135,136,137].

### 5.4. Analysis Using Artificial Intelligence (AI)

AI has substantially enhanced smartphone-based microfluidic analytical capabilities through automated interpretation of complex visual data patterns. Machine learning methodologies, particularly convolutional neural networks, demonstrate significant efficacy in classifying results from lateral flow assays, droplet configurations, and cellular imaging applications [138].

Deep learning models facilitate direct analyte concentration prediction from raw imaging data, eliminating manual calibration requirements. These approaches prove especially valuable for non-expert users in field deployment contexts, providing laboratory-quality analytical interpretation without specialized training prerequisites [139,140]. Transfer learning techniques address training data availability limitations, enabling model adaptation from related applications to specific forensic or agricultural implementation scenarios. AI algorithms analyze extensive datasets generated by microfluidic sensors, identifying trends and correlations not apparent through conventional statistical methodologies. Machine learning models undergo training for result classification, outcome prediction, and experimental condition optimization [141]. Real-time data analysis capabilities allow instantaneous user feedback, exemplified in agricultural applications where AI evaluates soil nutrient concentrations and provides fertilizer application recommendations based on sensor information [134]. Similarly, Liao et al. have developed a portable AI-driven microfluidic for algae monitor that integrates lensless imaging, neural networks, and touch interface, enabling automatic in-field water analysis [142]. It reduces size and cost while maintaining high accuracy, facilitating early environmental water pollution detection with improved portability over traditional lab equipment.

### 5.5. Commercial Smartphone-Based Microfluidic Sensors for Forensic, Agricultural, and Environmental Applications

Smartphone-integrated microfluidic sensors have transitioned from laboratory prototypes to commercial tools, offering portable, rapid, and accurate field-testing capabilities. In agriculture, devices such as smartphone-based lateral-flow assays have been used commercially to detect aflatoxin B1 in maize, employing phone cameras with macro lenses and LED illumination to enhance sensitivity and eliminate subjective readings [143]. RIDA^®^ SMART APP Mycotoxin (R-Biopharm AG, Darmstadt, Germany) is commercially available for detecting mycotoxins, including aflatoxin, deoxynivalenol, zearalenone, fumonisin, and Fusarium toxins T2 and HT-2, and is specifically designed for industry professionals and primary producers. The system employs a cover featuring a color reference chart and a QR code, which is positioned over the test strip before capturing the image. The entire assay takes 10 to 15 min to complete and does not require any large laboratory instruments or specialized expertise [144]. Environmental monitoring has also seen commercial gateways. Cell-phone-coupled microfluidic cytometers now enable the real-time detection, counting, and sizing of algal cells (e.g., in ballast water) with greater than 92% accuracy, using fluorescence imaging in combination with automated smartphone algorithms [103]. In the forensic realm, while purely commercial microfluidic forensic systems are still emerging, smartphone-enhanced portable microfluidic platforms have proven effective in reading optical signals using phone cameras to analyze blood or screen toxins, facilitating on-site sampling and analysis [145]. Collectively, these technologies illustrate how smartphone-based microfluidic chips are enabling decentralized, cost-effective, and immediate sensing solutions across diverse application domains.

The smartphone integrations on microfluidic sensors for forensic, agricultural, and environmental applications are summarized in Table 1, classified by the target molecule, sample type, sensing technique, chip fabrication method, smartphone integration, limit of detection (LOD), reaction time, and accuracy. Note that a small number of examples did not explicitly employ smartphones [146,147,148]. We nonetheless included them since they utilized equipment such as CMOS cameras, Bluetooth, etc., which are also available in smartphones.

## 6. Conclusions and Future Perspectives

The convergence of microfluidics and smartphone technologies has catalyzed a new era in portable diagnostics, offering low-cost, user-friendly, and field-deployable sensing platforms. This review critically evaluated smartphone-based microfluidic sensors across forensic, agricultural, and environmental domains, revealing both shared advantages and domain-specific nuances. Across all fields, these platforms significantly enhance on-site detection capabilities, minimizing reliance on centralized laboratories. Colorimetric assays, paper-based microfluidics (µPADs), and PDMS-based chips are the most dominant due to their simplicity, affordability, and compatibility with smartphone cameras. In agricultural and environmental monitoring, these devices primarily target pathogens, nutrients, pesticides, and water quality indicators using optical (colorimetric or fluorescent), electrochemical, and CRISPR-based approaches. In forensics, they address illicit drugs, explosives, and biomarkers in complex biological matrices (e.g., blood, urine, or saliva), often requiring higher sensitivity and specificity. Despite commonalities in fabrication and detection principles, application-specific constraints shape development trends. Forensic sensors often require ultra-low detection limits (e.g., ng/mL) and high specificity to minimize false positives in legal contexts. This necessitates advanced recognition elements (e.g., aptamers, nanomaterials, or enzyme–substrate systems) and increasingly leverages artificial intelligence or machine learning (AI/ML) algorithms for substance identification (e.g., convolutional neural network models for drug classification). Conversely, agricultural sensors tend to prioritize rapid, robust, and scalable detection of pathogens and chemicals in soil or water, where the sample matrices’ complexity is lower than in forensic samples.

Smartphone integration across all sectors has undergone rapid evolution. Beyond simple image acquisition, modern platforms support cloud connectivity, AI-driven data interpretation, wireless connections via Bluetooth, near field communication (NFC), or Wi-Fi, and integrated apps with geolocation and metadata tagging. Such capabilities not only support real-time decision-making but also enable traceability and regulatory compliance. Particularly promising is the integration of lensless imaging, quantum dots, and CMOS sensors, which offer high-resolution detection with minimal optical hardware requirements.

However, several challenges and limitations remain. Firstly, standardization is lacking. Differences in smartphone camera quality, lighting conditions, and chip materials can introduce significant variability in results. Calibration-free or self-calibrating systems, perhaps supported by on-board ML algorithms, are needed to ensure reliable cross-device performance. Secondly, reagent stability and shelf-life, particularly for enzyme- or nucleic acid-based assays, remain a concern in field deployment, especially under varying environmental conditions. In forensic applications, regulatory validation and evidentiary reliability are critical barriers to adoption. Devices must demonstrate reproducibility, chain-of-custody compatibility, and a strong correlation with gold-standard methods (e.g., GC-MS, HPLC). Agricultural tools face scalability issues, especially for smallholder farmers in low-resource settings. Environmental monitoring devices, while growing in popularity, still struggle with multiplexing and the dynamic range needed to detect diverse contaminants in water or soil simultaneously.

The future of smartphone-based microfluidic biosensors in forensic, agricultural, and environmental applications holds immense promise. Yet, it is intertwined with critical challenges that must be addressed to enable widespread implementation. One of the key future directions lies in the development of fully integrated lab-on-chip platforms that consolidate sample preparation, reaction, and detection into a single, miniaturized, and user-friendly system. Current approaches often rely on partially integrated systems, which require manual steps or peripheral instruments, limiting their portability and true point-of-need utility. Additionally, achieving standardized fabrication protocols for microfluidic chips across various materials (e.g., PDMS, paper, thermoplastics) and functions is essential to ensure reproducibility, scalability, and regulatory compliance, especially in sensitive sectors such as forensic testing. Another important area of advancement is the integration of advanced technologies such as CRISPR-based biosensing, AI-powered image processing, and wireless data transmission, which will enhance the specificity, speed, and usability of these sensors. For forensic applications, there is growing potential in integrating AI and ML algorithms to automate result interpretation, improving accuracy in substance detection (e.g., narcotics, DNA) while minimizing human bias. In agriculture, the deployment of multiplexed microfluidic systems capable of detecting multiple plant pathogens or agrochemicals simultaneously would improve crop management decisions and contribute to sustainable farming practices. In environmental monitoring, future sensors should be designed to detect emerging contaminants—such as microplastics, pharmaceuticals, and novel pathogens—with high sensitivity in complex sample matrices like soil and wastewater. Power autonomy and energy-efficient design are also crucial. Future devices should incorporate energy harvesting technologies (e.g., solar-powered chips or NFC-based sensors) to ensure long-term deployment in remote areas. Moreover, user-friendly interfaces and smartphone apps that support real-time data visualization, GPS tagging, and cloud-based storage will improve accessibility and facilitate large-scale epidemiological or ecological studies. Interoperability across platforms and compatibility with open-source data systems will also be crucial for enabling decentralized testing frameworks, especially in resource-limited or emergency contexts.

Finally, establishing regulatory frameworks and validation pipelines for these emerging diagnostic tools is imperative for their adoption in forensic casework, agricultural biosecurity, and environmental policy enforcement. With these strategic developments, smartphone-based microfluidic biosensors can revolutionize field diagnostics, making rapid, real-time, and precise sensing a reality across diverse and impactful domains.

## Figures and Tables

**Table 1:** Comparative summary of smartphone-based microfluidic sensors developed for forensic, agricultural, and environmental applications.

Ref.	Year	Target Molecules	Sample Type	Sensing Technique	Chip Fabrication	Smartphone Integration	LOD	Reaction Time	Accuracy
[149]	2014	Nitrite (NO_2_^−^)	Water	Colorimetric	Paper-based microfluidics; reagents deposited on patterned cellulose	Camera captures coloration; Custom app processes hue and saturation	0.52 mg/L	Rapid	-
[146]	2018	*Aspergillus niger* spores	Whole spores	Immunofluorescence using antibody-conjugated microspheres	PDMS-based chips (soft lithography)	No—fluorescence microscopy was used; Potentially adaptable for smartphone	300 spores/m^3^	2–3 h, incl. enrichment	>90%
[103]	2022	Live algae	Water	Optical detection	PDMS microchannel (soft lithography)	Camera captures images; Custom app counts algae	500 μm algae/s	Real-time	92%
[150]	2022	Nitrite (NO_2_^−^) and phosphate (PO_4_^3−^) ions	Soil samples	Colorimetric	PDMS-based chip (soft lithography) from UV-laser patterned mold	Smartphone powers the device; Bluetooth transfers data; app for near-range monitoring; cloud server for long-range access	0.33 µM NO_2_^−^; 0.75 µM PO_4_^3−^	2–3 min NO_2_^−^; ~10 min PO_4_^3−^	CV < 5%
[147]	2022	Microalga (*Scenedesmus quadricauda*)	Whole algal cells	Lensless imaging with CMOS sensor and neural network image segmentation	PDMS microfluidic chip (soft lithography); embedded with a lensless CMOS image sensor	No; suggested but not implemented; Bluetooth transfers data	-	Real-time	96.35%
[151]	2023	Iodide (I^−^) and Iodate (IO_3_^−^)	Seaweed extracts	Colorimetric	3D origami paper microfluidics (wax printing)	Camera captures the green channel intensity; ImageJ or app process images	9.8 µM I^−^; 0.6 µM IO_3_^−^	1 min	RSD 1.7% I^−^; 3.3% IO_3_^−^
[152]	2023	Mycotoxins AFB_1_, OTA, ZEN, FB_1_, T-2, and DON	Food samples	CRISPR/Cas12a system with quantum dots	PDMS (soft lithography)	Camera captures the fluorescence signals; images are analyzed via color model	1.4–3.9 fg/mL	40 min	CV < 5% 88.8–110% correlation with HPLC
[153]	2024	pH, ascorbic acid (AA), and 5-hydroxymethylfurfural (HMF)	Water samples	Colorimetric	Three-layer QR-coded paper microfluidics	Camera scans QR code and captures images; custom app quantify coloration	5.56 ppm AA; 6.73 ppm HMF; 0.1 pH units	10 min	CV < 10%
[86]	2024	Tomato pathogens: *Alternaria* spp., *Xanthomonas perforans*, *Phytophthora infestans*, tomato spotted wilt virus (TSWV)	Genomic DNA and RNA	Loop-mediated isothermal amplification (LAMP); fluorescence and colorimetric readouts	PDMS-based microfluidic chip (soft lithography) using a 3D-printed mold	Camera captures fluorescence images; ImageJ analyzes the images	1 pg DNA	30 min	90–100%
[154]	2025	Omethoate pesticide	Extracts from spinach, wheat, tap water	Aptamer-based fluorescence sensing	Laser-printed two-layered paper chip	Camera captures images; app with a CNN regression model quantifies fluorescence	0.16 nM	<10 min	R^2^ = 0.9964
[148]	2014	Nitrate, nitrite, chlorate, perchlorate, ammonium, TNT, RDX, PETN, TATP, urea nitrate, H_2_O_2_	Whole explosive compounds	Colorimetric	Wax printing on chromatography paper	Visual color changes assessed by eye or optionally scanned via color densitometry	0.39 to 19.8 mg	>10 min	-
[155]	2016	Cocaine and methamphetamine	Powdered drug samples	Colorimetric	Centrifugal microfluidic device from polyester-toner layers	Camera images reaction zones; images are analyzed via hue and saturation color spaces	0.25 (cocaine) and 0.75 (meth.) mg/mL	-	-
[156]	2016	Sweat biomarkers: chloride, lactate, glucose, pH	Sweat	Colorimetric	Soft, stretchable PDMS-based elastomer	Camera images detection zones; NFC transmits data	10–100 mM chloride	-	-
[157]	2017	Blood hematocrit	Whole blood samples	Colorimetric	Laser-cut polymer double-sided tape adhered to PMMA substrate	Camera captures blood images; app processes and analyzes images	0.1% hematocrit	1 min	Sensitive across 10–65% range
[158]	2017	Psychoactive drug: alprazolam (ALP)	Blood and vitreous humor	Colorimetric	Paper-based microfluidics with silver nanoparticles	Camera quantifies coloration	10 ng/mL	-	-
[159]	2018	Ethanol	Whole blood samples	Enzymatic detection (Aox and HRP)	Silicon microfabrication; Microfabricated Pt electrodes; Laser-cut PMMA	USB powers the device and acquire data from micro-potentiostat	0.0375 g/L	<5 min	Error < 0.009%
[160]	2018	Creatinine	Whole blood	Colorimetric	Paper-based microfluidics (wax printing)	Camera detects coloration; app processes data	-	5 min	-
[161]	2020	Human urinary creatinine	Urine	Colorimetric	Paper-based microfluidics (contact stamping)	Camera captures color images; ImageJ analyzes red-to-green (R/G) intensity ratio	-	-	RSD = 2.1% 98.1–104% correlation with HPLC
[162]	2024	Illicit drugs: cocaine, methamphetamine, MDMA, amphetamine, synthetic cathinones, pyrrolidine, methylenedioxy derivatives	Crushed or dissolved drug samples	Colorimetric	Microwell device (3D printing)	Camera captures colorations in microwells; RGB values processed using an artificial neural network (ANN)	μg range	<5 min	>83.4% sensitivity; 100% specificity
[54]	2024	Ethanol and Δ^9^-THC	Saliva	Colorimetric	Paper-based microfluidics (wax printing)	Camera mages detection zones; app quantifies colorations	-	40 min	Recovery 98–102% ethanol and 95–105% THC; RSD < 5%
